# Western Dental Society

**Published:** 1867-04

**Authors:** 


### Proceedings of Societies.

### WESTERN DENTAL SOCIETY.

The Western Dental Society held its eleventh annual meeting in the infirmary of the Missouri Dental College, St. Louis, Mo., 12 o'clock, M., January 9, 1867.

The President, Dr. T. P. Abell of Chicago, being absent, the meeting was called to order by 1st Vice President Dr. C. W. Rivers, Tittsville, Ill.

Proceedings of the meeting in 1865 read and approved.

1. Dr. H. E. Depp of Sedalia, Mo., proposed for membership.

2. Treasurer's report.

Chairman of the Microscopic Committee, reported no progress. Report received and committee discharged.

3. The election of officers being in order, the following named gentlemen were elected for the ensuing year.

President—Dr. C. W. Rivers, St. Louis, Mo.
1st. Vice President—W. H. Evans, D. D. S. "
2d. Vice President—Dr. E. Hale, jr., "
Recording Sec'y—W. N. Morrison, D. D. S. "
Corresp'ng Sec'y—D. A. W. French, Springfield, Ill.
Treasurer—H. E. Peebles, D. D. S., St. Louis, Mo.

Dr. S. L. Edwards, Griggsville, Ill.; M. McElroy, Booneville, Mo.; C. W. Rivers; A. W. French; E. Hale, jr., Executive Committee.

Adjourned to meet at the same place at 2½ o'clock, P. M.

### AFTERNOON SESSION.

Executive Committee reported favorably in the case of Dr. Depp, who upon ballot was duly elected a member.

The Secretary reported a list of those who were delinquent in the payment of dues.

On motion of Dr. Edwards, the annual dues for the years 1866 and 1867, were abolished.

The executive committee reported that they approved of so much of the late Treasurer's—Dr. C. W. Spaulding—report as relates to advertising, moving chairs, Janitor's fee, etc.

The balance, $190 39, which report says was given or loaned to Professor George Watt, the committee respectfully submit should be paid to the treasurer elect, Dr. Peebles, to be held subject to the order of the Society.

Dr. McCoy moved that when we adjourn we adjourn *sine die*, and that any funds remaining in the hands of the treasurer, be paid over to the Missouri Dental College.

Adjourned to 10 o'clock, A. M., to-morrow.

### SECOND DAY, MORNING SESSION.

President in the chair. Minutes of last meeting read and approved.

Money in treasury $190 39. Circulars, advertising, &c., $7 00, leaving a balance in treasury of $183 39.

On motion of Dr. A. Blake, the funds remaining in the treasury be divided equally between the Ohio and Missouri Dental Colleges. Unanimously adopted.

The following named gentlemen were elected delegates to the American Dental Association, viz: Drs. A. W. French, H. E. Depp, M. McCoy, I. Comstock, W. A. Cornelius, C. W. Rivers, J. Ward Ellis.

After a vote of thanks to retiring officers, Dr. Peebles moved that we adjourn to meet at the call of the President.

Adjourned. W. N. MORRISON, Sec'y.

